# GT Transcription Factors of *Rosa rugosa* Thunb. Involved in Salt Stress Response

**DOI:** 10.3390/biology12020176

**Published:** 2023-01-22

**Authors:** Jianwen Wang, Yufei Cheng, Xinwei Shi, Liguo Feng

**Affiliations:** College of Horticulture and Landscape Architecture, Yangzhou University, Yangzhou 225009, China

**Keywords:** *Rosa rugosa* Thunb, GT or trihelix, halophytes

## Abstract

**Simple Summary:**

Salt stress produced ion toxicity on plant cells and limited the of culture of cultivated *Rosa rugosa*. GT genes in salt stresses responses have been emerging. From the *GT* gene family of the salt-tolerant wild *Rosa rugosa,* four NaCl stress responsive genes (*RrGT-1, RrSIP1, RrSIP2, RrGTγ-4*) were identified. *RrSIP1* and *RrGTγ-4, RrGT-1* and *RrSIP2* located on chloroplasts and cell nucleus, respectively. *RrSIP1, RrSIP2* and *RrGTγ-4* could play roles in regulation of sodion and potassium transport. And *RrGT-1* expressed higher specifically in wild *Rosa rugosa* than in the salt-sensitive cultivated *Rosa rugosa*. These four genes would be candidates for further study of regulation mechanism of salt-tolerance of wild *Rosa rugosa* and would supply gene resources for tolerance improvement of cultivated *Rosa rugosa.*

**Abstract:**

*Rosa rugosa* was a famous aromatic plant while poor salt tolerance of commercial cultivars has hindered its culture in saline-alkali soil. In many plants, the roles of *GT* (or *trihelix*) genes in salt stresses responses have been emerging. In the wild *R. rugosa,* a total of 37 *GTs* (*RrGTs*) were grouped into GT-1, GT-2, GTγ, SH4, and SIP1 lineages. SIP1 lineage expanded by transposition. The motifs involved in the binding of GT cis-elements were conserved. Four *RrGTs* (*RrGT11/14/16/18*) significantly differentially expressed in roots or leaves under salt stress. The responsive patterns within 8 h NaCl treatment indicated that *RrGTγ-4* (*RrGT18*) and *RrGT-1* (*RrGT16*) were significantly induced by salt in roots of *R. rugosa*. Subcellular localizations of *RrSIP1* (*RrGT11*) and *RrGTγ-4* were on chloroplasts while *RrGT-1* and *RrSIP2* (*RrGT14*) located on cell nucleus. Regulation of ion transport could be the most important role of *RrSIPs* and *RrGTγ-4*. And *RrGT-1* could be a halophytic gene with higher transcription abundance than glycophytic *GT-1*. These results provide key clue for further investigations of roles of *RrGTs* in salt stress response and would be helpful in the understanding the salt tolerance regulation mechanism of *R. rugosa*.

## 1. Introduction

GT (or trihelix) family is a plant-specific transcription factor (TF) family with a DNA-binding domain rich in proline and glutamine residues [1,2]. The conserved DNA-binding domain containing three tandem helix structures (helix-loop-helix-loop-helix), namely trihelix domain [1,2]. The highly similar structure of trihelix domains and Myb/SANT-LIKE DNA-binding domains indicated that GT factors evolved from Myb/SANT-LIKE proteins [3]. GT elements are photosensitive cis-elements with A/T-rich motifs like ‘(T/A) -A- (T/A)’ [4]. Interactions between GT factors and GT elements are implicated in the light dependent expression of many plant genes. e.g., the first identified *GT* gene *GT-1* binds two light-responsive elements of *Rubisco small subunit 3A (rbcS-3A)* of *Pisum sativum* L. [5]. A total of 30 *GT*s in *Arabidopsis thaliana* (L.) Heynh (*AtGTs*) were identified and classified into GT-1, GT-2, GTγ, SH4, and SIP1 lineages [6]. Most *AtGTs* played complex transcriptional regulation roles in light-responsive gene regulation [7], immunity (*ARABIDOPSIS SH4-RELATED3*, *ASR3*) [8], perianth architecture regulation and fertility (*PETAL LOSS, PTL*) [9] and seed maturation (*Arabidopsis 6b-interacting protein 1-like 1*, *ASIL1*) [6]. Recently, roles of abiotic stress responses were identified in *GT* family, like *AtGTs* involved in salt-induced gene expression (*Arabidopsis GT-1-like transcription factor*, *GT-3A*) [10], hypoxia-responsive response (*AT3G10040*) [11] and salt and/or other abiotic stress tolerances (*Arabidopsis SIP1 clade Trihelix1*, *AST1; GT-4, GT2L*) [12,13,14]. Salt stress produced osmotic stress and ion toxicity on plant cells, resulting in the destruction of organelles and excess accumulation of activated oxygen [15,16]. Further, the photosynthesis decreasing and poor growth under salt stress contributed to the losses of quality and yield of economic crops [17]. The expression profile studies predicted some *GTs* which responded to salt stress in many crops, like cotton [18], wheat [19], *Brassica napus* L. [20], quinoa [21], *Sorghum bicolor* L. [22], soybean [23] and rice [24]. Function studies have proved salt tolerance regulation roles of several crop *GTs.* e.g., *OsGTγ-1* (*Os02g33770*) and *OsGTγ-2* (*Os11g06410*) of rice positively regulate salt tolerance [24]. *GmGT-2A*, *GmGT-2B* of soybean [25] and *GhGT26* of cotton [26] enhance salinity tolerance in overexpressed Arabidopsis.

Salinity stress is one of the most severe abiotic stresses and poses a continuing threat to economic crops [27], and for this reason the research are starting to consider and valorize wild species suitable for saline environments [28,29]. *Rosa rugosa* cultivars are widely used for spicery of food industry or essential oil of cosmetics industry [30,31]. These *R. rugosa* cultivars lost salt tolerance along with the breeding processes of floral traits, resulting in their limiting planting areas although there were vast saline-alkali soils in China [32,33,34]. e.g., the commercial cultivar *R. rugosa* ‘Zizhi’ (Zizhi) which planted in the narrow hilly lands of Shandong Province (China) was a typical glycophyte. While the wild *R. rugosa* which distributed naturally in the coastal area of northeast China belong to halophytes. The wild *R. rugosa* kept strong salt tolerance to adapt to the high salinity beach, as observed in other plant species in the coastal areas in the world [35]. In genetic engineering of salt-tolerance for glycophytic crop, homologous genes from halophytes should be more efficient since halophytes are more salt-tolerant than glycophytes [28,36]. The mining of salt responsive TF of wild *R. rugosa* would supply a base for salt tolerance improvement of *R. rugosa* cultivars [34].

With the genome of wild *R. rugosa,* this study aimed to screen *GTs* of *R. rugosa* (*RrGTs*) involved in the salt response. The phylogeny, synteny and sequence analyses would give a systematic understanding of lineage, gene duplication events, conserved motifs and gene structures of *RrGT* family. Expression profiles of salt treated *R. rugosa* were built to detect significantly induced/reduced *RrGTs*. These candidates were pointcuts of further study of salt stress responses regulation. Our study preliminary studied the roles of *RrGTs* under slat stress and would be helpful in understanding the regulatory mechanism of salt tolerance of *R. rugosa*.

## 2. Materials and Methods

### 2.1. Identification of RrGT Family

The *R. rugosa* and *Rosa chinensis* Jacq. genomes were obtained from GDR (Genome Database for Rosaceae, https://www.rosaceae.org/, accessed on 1 May 2022). Based on the hidden Markov model Myb/SANT-like DNA-binding domain (PF13837, http://Pfam.sanger.ac.uk/, accessed on 1 May 2022), candidate proteins were screened from the genome using HMMER 3.3.2 [37] with a cutoff threshold value of E-5. The ambiguous or incomplete candidates were rejected by TF verification of PlantTFDB (http://planttfdb.cbi.pku.edu.cn/, accessed on 1 May 2022).

### 2.2. Phylogenetic Analyses of RrGT Proteins

*GT* genes of *Oryza sativa* (*OsGTs*) and *Arabidopsis thaliana* were obtained from PlantTFDB (http://planttfdb.cbi.pku.edu.cn/, accessed on 1 May 2022). Based on the alignment of GT domains using MAFFT 7.55 (https://mafft.cbrc.jp/alignment/server/, accessed on 1 May 2022), a neighbor-join (NJ) phylogenetic tree was constructed by MEGA 7 with 1000 bootstrapping replications [38]. Besides, the p-distance model of the substitution type, pairwise deletion of Gaps/Missing data and uniform rates among sites were selected for the phylogenetic analysis.

### 2.3. Synteny Analysis of RrGTs

The homologous gene pairs (*E* < 10^−5^, top five matches) within the *R. rugosa* genome or among *R. rugosa*, *R. chinensis* and *Fragaria vesca* L. genomes (obtained from GDR) were identified by BLASTP (BLAST+ 2.13.0) search. Based on the location of homologous pairs, MCScanX (mcscan2) [39] identified the syntenic regions and predicted the gene duplication events. *RrGTs* and corresponding homologous *GTs* on syntenic regions were highlighted by Synteny plot tool of TBtools [40].

### 2.4. Gene Structure, Motif Analysis and Cis-Acting Elements of RrGT Family

The top 10 conserved motifs were predicted by MEME web tools (https://meme-suite.org/meme/, accessed on 2 May 2022) under default parameters. Cis-elements on the 2000 bp sequence upstream to the initiation codon were predicted by PLANTCARE (http://bioinformatics.psb.ugent.be/webtools/plantcare/html/, accessed on 2 May 2022). The gene structures and motifs were illustrated by the Gene structure view tool of TBtools [40].

### 2.5. Expression Analysis under Salt Stress

Previous transcriptome data [32,41] of wild *R. rugosa* and *R. rugosa* ‘Zizhi’ provided the per kilobase of exon model per million mapped fragments (FPKM) of *RrGTs* and the fold-changes of differentially expressed *RrGTs.*

One-month-old wild *R. rugosa* seedlings were treated with 340 mM NaCl solution for 0.5 h, 1 h, 2 h, 4 h and 8 h and roots of these samples were collected with three biological repetitions. Total RNAs were extracted and reverse-transcribed as cDNA templets by RNAprep Pure plant kit (Tiagen, Beijing, China) and HiScript^®^ III RT SuperMix (Vazyme, Nanjing, China). Quantitative real-time polymerase chain reaction (qRT-PCR) was conducted using ChamQ SYBR Color qPCR Master Mix (Vazyme, Nanjing, China) on the CFX96 platform (Bio-Rad, China). All the steps of RNA extraction, reverse-transcription and qRT-PCR were conducted following the manufacturers’ recommended instructions. Table S5 listed the primers of *RrGTs* and reference genes (phD and 5.8s).

### 2.6. Subcellular Localization Analysis

The subcellular localization of *RrGTs* were predicted by webtools Plant-mPLoc (http://www.csbio.sjtu.edu.cn/bioinf/plant-multi/, accessed on 2 May 2022) and WoLF PSORT (https://wolfpsort.hgc.jp/, accessed on 2 May 2022). Coding sequences of 4 candidate *RrGTs* were cloned into 35S: green fluorescent protein (GFP) vector pNC-AMP-GFP-C and transformed to Arabidopsis protoplasts for overexpression of RrGT-GFP fusion proteins. The fluorescence of chloroplasts, GFP and 4′,6-diamidino-2-phenylindole (DAPI, Nuclear marker) were observed by the laser confocal microscopy.

## 3. Results

### 3.1. Lineages and Synteny of RrGT Family

**Figure 1:**
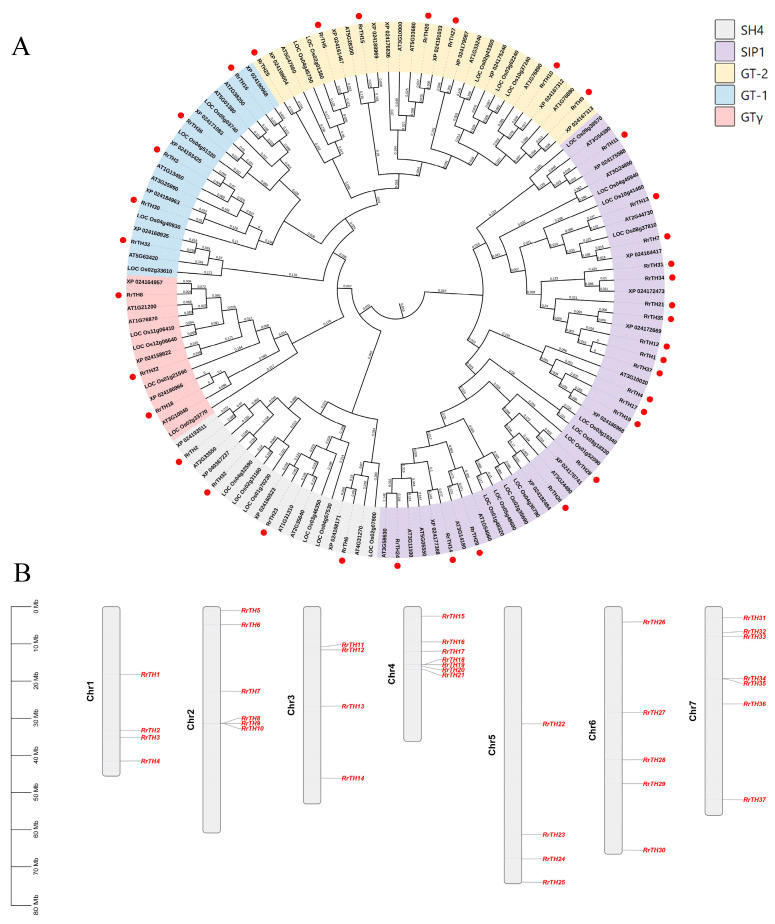
The phylogeny (**A**) and chromosome location (**B**) of *GT* family of *Rosa rugosa* (*RrGT*). The dendrogram of *GTs* of *R. rugosa*, *Rosa chinensis* (XP), *Oryza sativa* (LOC) and *Arabidopsis thaliana* (AT) was generated using the neighbor-joining method with 1000 bootstrap replicates (numbers in branches). Nodes were colored according to the five lineages of *GT* family and *RrGTs* were highlighted by red dots. The gene ID was listed in Table S2.

A total of 37 *RrGTs* scattered across the seven chromosomes of *R. rugosa* genome (Figure 1B, Table S2). The NJ-tree of *RrGTs, OsGTs* and *AtGTs* divided 18, 7, 5, 4, 3 *RrGTs* to SIP1, GT-2, GT-1, SH4 and GTγ lineages, respectively (Figure 1A). The gene number of *RrGT* of each linage is similar to that of *OsGTs* or *AtGTs* of corresponding lineage except SIP1 (Table S1).

**Figure 2:**
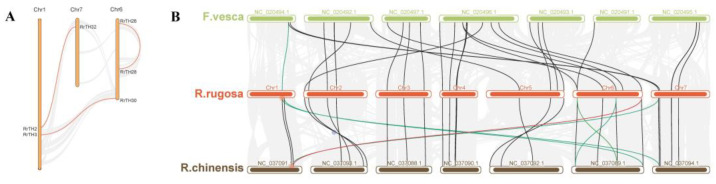
The intra-species (**A**) and inter-species (**B**) synteny of *RrGT* family. (**A**) The paralogous *RrGTs* on synteny regions (dark lines) of chromosome 1/6/7. Three *RrGT* pairs are highlighted (Table S2). (**B**) Synteny regions (dark lines) of genomes of *R. rugosa* (red), *Fragaria vesca* (green), *R.chinensis* (brown) and orthologous *GTs* (color lines). The orthologous *GT* pairs are linked by black lines. Green lines indicate *GT* pairs on overlapped synteny regions.

WGD (whole genome duplication) or segmental duplication produced three paralogous *RrGT* pairs, namely *RrTH2-RrTH32, RrTH3-RrTH30*, *RrTH26-RrTH28* on intra-species synteny regions of Chr1-Chr7, Chr1-Chr6 and Chr6-Chr6, respectively (Figure 2A, Tables S2 and S3) and 22 orthologous gene pairs of *RrGTs-RcGTs (GTs* of *R. chinensis)-FvGTs (GTs* of *F. vesca)* on inter-species synteny regions among *R. rugosa*, *R. chinensis* and *F. vesca* (Figure 2B, Table S2). Other *RrGTs* were predicted as ‘dispersed type’ which might arise from transposition and no tandem duplication events were identified. *RrTH12, RrTH30*, *RrTH32* produced five redundant homologous pairs on the synteny regions of non-homologous chromosomes (Figure 2B, red and green lines). After removing the small synteny regions which including less than 20 gene pairs (Figure S1), only one redundant pair was credible (Figure 2B, red line). The consistent collinearity indicated that the lineage evolution of *GT* families among *R. rugosa*, *R. chinensis* and *F. vesca* was conserved (Figure 2B).

It was worth noting that 7 *RrTHs* (*RrTH1/4/12/17/21/31/37*) of SIP1 lineage and *RrTH10* of GT-2 lineage lacked homologous *GTs* in synteny regions (Table S2). All these *RrTHs* in SIP1 lineage were dispersed type and it indicated transposition should contributed to the SIP1 lineage expansion of *R. rugosa*.

### 3.2. Gene Structures and Conseved Motifs of RrGT Family

**Figure 3:**
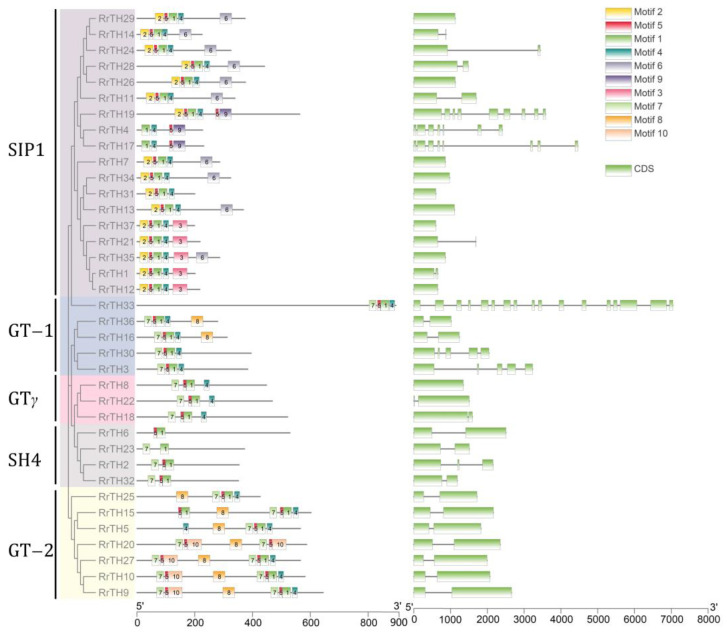
The conserved motifs and exon–intron structures of *RrGT* family. Five lineages are indicated by colored branches of NJ-dendrogram. The top 10 conserved motifs (boxes with numbers) were located by amino acid scale plate. The exons (green boxes) and introns (lines) were located by the nucleotide scale plate.

The length of RrGT proteins varied from 199 to 896 amino acids (aa), with a molecular weight range of 21.977 (*RrTH37*) to 99.117 kDa (*RrTH33*). RrGT proteins of GT-2 lineage were longer due to their two trihelix domains. And the N-terminal trihelix domain (corresponding to the ‘7-5-10’ motif group) was spaced from the C-terminal trihelix domain by motif 8. In other linages, ‘2-5-1-4’ motif group or ‘7-5-1-4’ motif group constituted the trihelix domains (Figure 3, Table S4). Besides, motif 6, motif 9, motif 3 and motif 8 were specific to SIP1 lineage and GT-1/GT-2 lineage, respectively.

Over half *RrGTs* (20 *RrGTs*, 54.05%) included 2 exons and 10 *RrGTs* (27.03%) were intron-free. One *RrGT* contained 3, 7, 8 and 9 exons and two *RrGT* genes contained 5 exons, respectively. Exons of *RrGT33* were up to 17 but most (exon 1–exon 12) coded the long nonconservative N-terminal with no conserved motifs.

### 3.3. Expression Analysis of RrGTs

**Figure 4:**
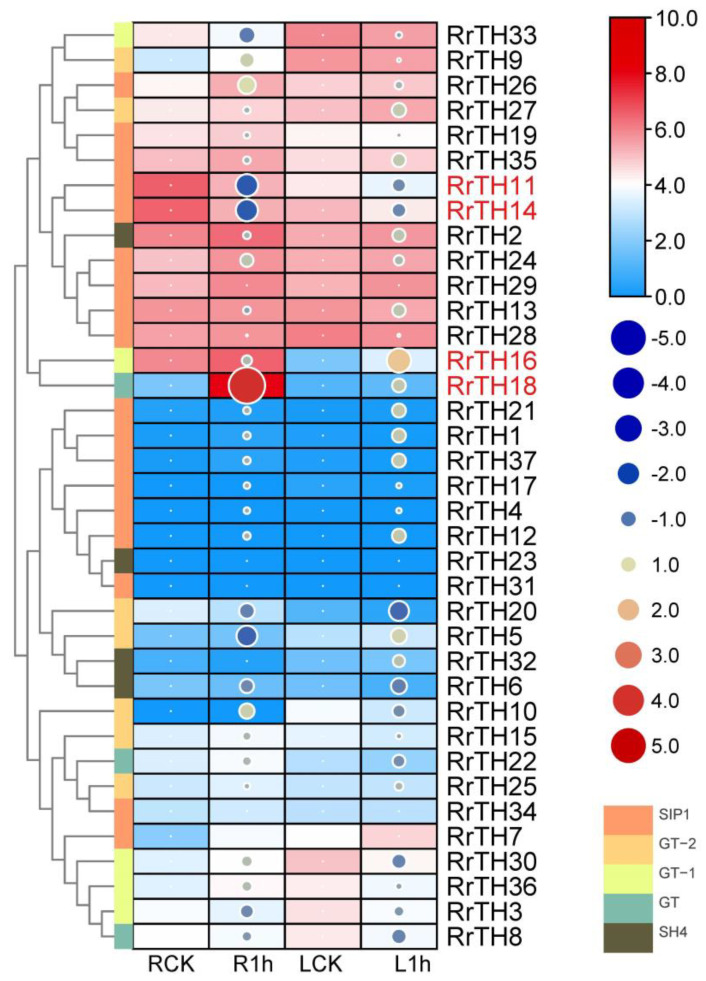
Expression profiles of *RrGTs* under salt stress in roots and leaves of *R. rugosa*. Four columns indicated roots or leaves of *R. rugosa* seedings treated by NaCl solution (R1h or L1h) and water (RCK or LCK) for 1 h. Normalized FPKM (per kilobase of exon model per million mapped fragments) (Table S2) are indicated by the boxes with gradient colors. Log2(fold-change) (Table S2) using RCK and LCK as controls were indicated by size and gradient color of circles. Rows were clustered by hierarchical clustering of Euclidean distance. Five lineages were represented by colored nodes of clustergram. Differentially expressed *RrGTs* were labeled in red.

In the RNA-seq expression profiles of wild *R. rugosa* under salt stress, 15 and 10 *RrGTs* were clustered as high- and middle-abundance genes, respectively (Figure 4, hierarchical clustering). In the 12 low abundant genes, except *RrGT5/6/25/32* (10 > FPKM > 1) belonging to GT-2 and SH4 lineages, other 8 extremely low abundant *RrGTs* (FPKM < 1) were corresponding to the SIP1 lineage *RrGTs* without corresponding collinear *RcGTs* and *FvGTs* (Table S2).

Four differentially expressed genes were predicted as salt responsive *RrGTs* (Figure 4, red labeled *RrGTs*). Under salt stress, *RrTH18* and *RrH16* upregulated 16.19 fold in roots and 3.47 fold in leaves, respectively. *RrTH11* and *RrTH14* downregulated 0.35 fold, 0.36 fold in leaves, respectively (Table S2). The four candidate genes *RrTH18, RrTH11, RrTH14* and *RrH16* were named as *RrGTγ-4*, *RrSIP1*, *RrSIP2* and *RrGT-1*, respectively.

**Figure 5:**
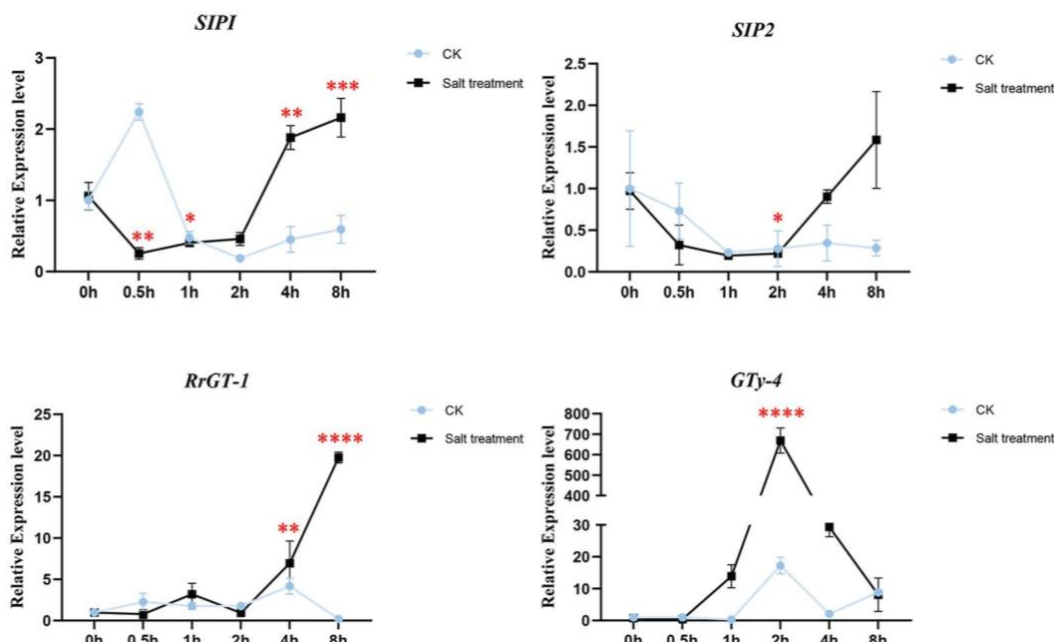
The salt responsive expression levels of *RrGTγ-4*, *RrSIP1*, *RrSIP2* and *RrGT-1* in roots of *R. rugosa*. The wild *R. rugosa* seedings were treated by water (CK) and 340 mM NaCl solution (Salt treatment) from 0.5 h to 8 h. Untreated seedings were the reference samples (0 h). *, **, *** and **** indicated the threshold value of significance of *t*-test 0.05, 0.01, 0.001 and 0.0001, respectively.

The expression patterns of the four candidate genes were checked in wild *R. rugosa* seedings treated by water (CK) and 340 mM NaCl solution (Figure 5). The salt responsive expression of *RrSIP1* was down-regulated in 0.5 h, kept low abundance in 1 h and 2 h, then significantly up-regulated in 4 h and 8 h. Interestingly, water stress (CK) induced significant upregulation in 0.5 h and downregulation after 0.5 h of *RrSIP1* which was opposite to its salt stress response. The expression of *RrSIP2* only significantly downregulated in 2 h salt stress. The expression of *RrGT-1* was stable before 2h then significantly upregulated to 19.08 fold in 8 h. *RrGTγ-4* responded to salt stress tempestuously and rapidly. Its expression significantly upregulated to 14.5 fold from 1 h, reached 669 fold in 2 h then downregulated to 8.2 fold in 8 h. Water stress also induced the upregulations of *RrGTγ-4* but the fold changes were far from inducement of salt stress.

### 3.4. Subcellular Localization of RrGT Candidates

The predicted subcellular localizations (Table S2) of *RrGT* family were diverse (Nuclei, chloroplasts, mitochondria, cytoplasm and so on). And different prediction tools were inconsistent in prediction of several *RrGT*s. e.g., *RrSIP1* and *RrSIP2* were predicted as proteins located on nucleus by Plant-mPLoc but on chloroplasts by WoLF PSORT. We detected the subcellular localizations of above 4 candidates (*RrGTγ-4*, *RrSIP1*, *RrSIP2* and *RrGT-1)* to exclude the inconsistent prediction.

**Figure 6:**
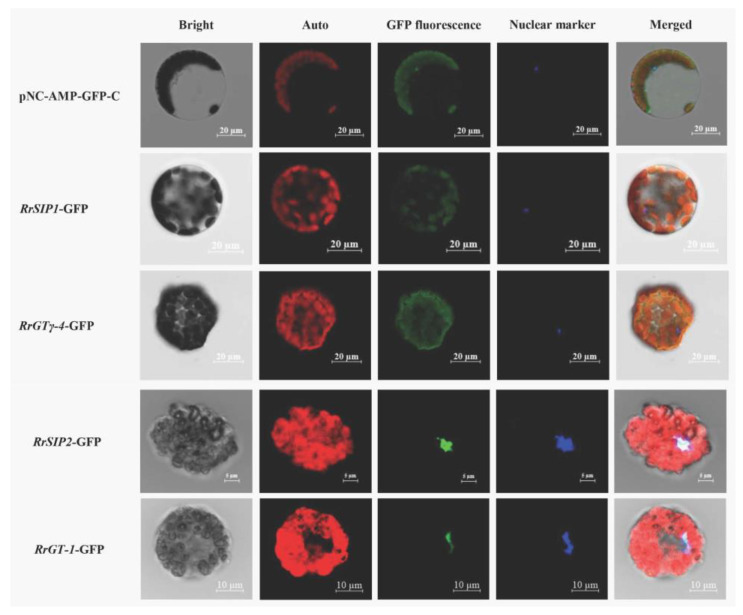
The subcellular localizations of *RrGTγ-4*, *RrSIP1*, *RrSIP2* and *RrGT-1*. Arabidopsis protoplasts transformed by GFP empty vector (pNC-AMP-GFP-C) or 35S: *GTs*-GFP vectors were observed using the laser confocal microscopy. The merged figures (Merged) were based on bright fields (Bright) and fluorescence of chloroplasts (Auto), green fluorescent protein (GFP), 4’,6-diamidino-2-phenylindole (DAPI, Nuclear marker) on dark fields.

The fusion proteins of RrSIP1-GFP or RrGTγ-4-GFP (green) overlapped with chloroplasts (red) indicated that *RrSIP1* and *RrGTγ-4* were located on chloroplasts. The colocalizations (cyan) of fusion proteins (green) and nuclear marker (blue) indicated that *RrGT-1* and *RrSIP2* were located on nucleus (Figure 6).

## 4. Discussion

### 4.1. The Species Specific Expansion of SIP1 Lineage

Expansion of SIP1 lineage has been observed in the *Brassica* plants. The majority of SIP1 genes of *Brassica rapa* L. retained two or three copies of corresponding *AtGTs* and it was more than other four lineages (one copy) [42]. The *Brassica* specific whole genome triplication contributed to the SIP1 lineage expansion since its divergence from *A. thaliana* [42,43]. But the conserved number of SIP1 genes of *R. rugosa* relative plants (8 *GTs* of *F. vesca* and 10 *GTs* of *R. chinensis*) indicated genome triplication or WGD did not contribute to *R. rugosa* SIP1 lineage expansion, and the specific expansion happened after the divergence from *R. chinensis* (Table S1). The 7 *RrGTs* (*RrTH1/12/17/21/31/37*) without corresponding collinear *RcGTs* and *FvGTs* were predicted as dispersed duplication types, which indicated proximal duplication and tandem duplication did not contribute to the SIP1 lineage expansion. And 5 of above genes (*RrTH1/12/21/31/37)* were clustered into the *Rosa*-specific lineages of the dendrogram with *RrTH34/35* and transcript abundances of the 5 genes were extremely low in roots and leaves (Figure 4). These observations indicated part of *RrGTs* in SIP1 lineage could arise from transposition, which contributed to SIP1 lineage expansion of *R. rugosa*.

### 4.2. Salt Responsive Candidates of RrGTs

In GT-1 lineage, *GT-3A (AT5G01380)* binds to GT elements of salt-induced *SCaM-4* gene (Ca2+-binding protein) of Arabidopsis and soybean [10]. The interaction of *GT-4* (*AT3G25990*) and *TEM2* (a B3 and AP2/ERF domain-containing protein) improved Arabidopsis salt tolerance by activating *Cor15A (AT2G42540)* [14]. *RrGT-1* clustered with *GT-3A* and *AT2G38250* (no reports on abiotic stress) in same cluster of GT-1 lineage (Figure 1). *RrGT-1* expressed much higher in roots (FPKM = 60.15) than in leaves (FPKM = 2.5). *RrGT-1* upregulated within 1 h salt stress only in leaves but strongly upregulated in roots under 8 h salt stress. The inconsistent inducing timing indicated *RrGT-1* responded to salt more promptly in leaves than in roots.

In *GTγ* lineage, *HRA1 (HYPOXIA RESPONSE ATTENUATOR 1, AT3G10040)* attenuates the anaerobic response induced by ERF-VIIs by protein interaction with RAP2.12 [11]. In rice, three *OsGTγ* genes were induced by salt, abscisic acid (ABA) or other abiotic stresses [24,44]. *OsGTγ-1* was significantly induced by salt and its mutant increased salt stress sensitivity while *OsGTγ-1* overexpression enhanced salt tolerance [24]. Similar, knockout and overexpression of *OsGTγ-2* increased salt stress sensitivity and tolerance, respectively. In our study, *RrGTγ-4* clustered with *OsGTγ-1* and *HRA1* in same cluster of *GTγ* lineage. *RrGTγ-4* only highly expressed in roots under salt stress (FPKM = 271.54) and its abundance was much higher than other *RrGTs* (FPKM < 83). qRT-PCR proved the rapid and dramatic salt-inducing of *RrGTγ-4* in roots within 1 h and peaked at 2 h, which was similar to *OsGTγ-1. RrGTγ-4* should be a key candidate involved in salt tolerance regulation of *R. rugosa* roots.

In *SIP1* lineage, *NtSIP1* was firstly identified from Agrobacterium 6b-interacting proteins of *Nicotiana tabacum* L. [45]. *AST1* (*At3g24860)* binds to a Novel AGAG-Box of stress tolerance genes to regulate Arabidopsis salt tolerance positively [12]. *BnSIP1-1* overexpression improved both osmotic and salt stresses tolerance during seed germination, but only improved osmotic stress tolerance of transgenic *B. napus* plants [20]. In our study, *RrSIP1, RrSIP2* were highly expressed in roots and leaves (FPKM > 20) and downregulated under salt stress only in leaves. Though most studies focus on stress induced *GT* genes, the two salt-stress-reduced *RrGTs* could be candidates involved in the negative regulation of salt tolerance.

Though no candidate *RrGTs* of GT-2 lineage significantly induced or reduced by salt, some studies have indicated GT-2 lineage also played roles in stress responses. e.g., Arabidopsis *gtl1* (*GTL1, AT1G33240*) mutant improved water use efficiency by reducing leaf transpiration [46], and *GT2L (AT5G28300)* induced significantly by salt, drought, cold and ABA and it responded to cold and salt stresses by interaction with calcium/calmodulin [13].

### 4.3. Potential Target Genes and Regulation Roles of RrGTs

Previous study indicated that *R. rugosa* responds to ion stress by gene expression regulation but rather gene dosage since its ion transporter gene number was conserved [34]. In rice, *OsGTγ-2* directly interacted with the GT-1 element ‘GAAAAA’ of three ion transporter genes (*OsHKT2; 1, OsNHX1* and *OsHKT1; 3)* [44]. In the ion transporter genes of the *R. rugosa,* GT-1 elements were found in the promoters (1000 bp upstream) of *RrHKT (evm.model.Chr5.2560,* only one *HKT* in the genome) and 6 of all 8 *RrNHXs (evm.model.Chr1.2289, evm.model.Chr1.4465* which is the only one *RrSOS1* [34], *evm.model.Chr2.1941*, *evm.model.Chr2.1942*, *evm.model.Chr4.416* and *evm.model.Chr5.7017*). Most transporter genes contained 1-4 GT-1 elements (-300 to -100 mostly) while only *evm.model.Chr5.7017 (AtNHX5/6* homolog) identified 7 GT-1 elements all across the promoter (−700 to −100). It indicated that *RrGTγ-4* could be regulator of these ion transporter genes and coordinated ion transport under salt stress.

*SIPs* mostly take part in ABA signaling to resist growth inhibiting effect of salt stress. In apple, *MdSIP1-2* promoted lateral root development promotion which was associated with ABA sensitivity, drought and salt stress tolerance [47]. *AST1* and *BnSIP1-1* reduced water loss rat in the overexpressed seedings [12,20]. The osmotic stress marker genes were highly expressed both in the *BnSIP1-1* transgenic plants and seedings whereas ion transporter genes (*BnSOS1, BnNHX1*, and *BnHKT*) were only significantly higher expressed in transgenic seedings (not in plants). *SIP* genes seem to exert different regulatory mechanisms along with different development process. Interestingly, the AGAG-Box ‘GGTAAA’ was found in two *RrNHXs (evm.model.Chr5.2640, evm.model.Chr4.534)* which lacked GT-1 elements. It indicated that excess ion response and plant developing regulation were crossed in *R. rugosa* by *RrSIPs*.

The genes of halophytes might be more superior for plant growth in saline-rich areas than their orthologs of glycophytes [28,29]. The 4 *RrGT* candidates were homologous to corresponding glycophytic *GTs* of Zizhi (over 90% protein identity). In roots, only *RrGT-1* (FPKM = 60.15, this study) expressed significantly higher than *GT-1* of Zizhi (FPKM = 2.34 [41]). *RrGT-1* could be a halophytic gene with higher transcription levels in wild *R. rugosa*. While the post translational regulation not transcription contributed mostly of salt-tolerance difference between halophytes and glycophytes. Whether the *GTs* were involved in the post translational regulation was not studied yet and it could be an important way to study the roles of other 3 *RrGTs* specific to halophytes [28,29].

## 5. Conclusions

In this study, we identified the *RrGT* family with 37 members from the *R. × rugosa* genome. *RrGTs* belonged to 5 lineages and SIP1 lineage expanded significantly. The conserved motif groups corresponding to trihelix domains were most conserved. *RrGTγ-4* (*RrGT18*), *RrGT-1* (*RrGT16*) located on chloroplasts and *RrSIP1* (*RrGT11), RrSIP2* (*RrGT14)* located on nucleus. The four genes significantly differentially expressed under salt stress in roots or leaves. Regulation of ion transport could be the most important role of *RrSIP* genes and *RrGTγ-4* in response to salt stress of wild *R. rugosa*. And *RrGT-1* could be a halophytic gene with higher transcription abundance than glycophytic *GT-1*. Together, ion transport regulation roles of GT needed to be illuminated and the regulation role of GTs specific to halophytes would be a breakthrough point for further researches. 

## Data Availability

Not applicable.

## Supplementary Materials

The following supporting information can be downloaded at: https://www.mdpi.com/article/10.3390/biology12020176/s1. Figure S1: The synteny analysis of *RrGTs, RcGTs* and *FvGTs*; Table S1: Numer of GT genes of different plants; Table S2: Gene information, synteny analysis by MCscanX, RNA-seq and prediction of subcellular localizations of *RrGTs*; Table S3: The intra-species synteny regions; Table S4: Top 10 conserved motifs of *RrGT* family; Table S5: Primers for qRT-PCR.
