# Supplementary figures and images for: GT Transcription Factors of Rosa rugosa Thunb. Involved in Salt Stress Response
Source: Biology (Basel). 2023 Jan 22;12(2):176. doi: 10.3390/biology12020176 (PMC9952457; doi:10.3390/biology12020176)

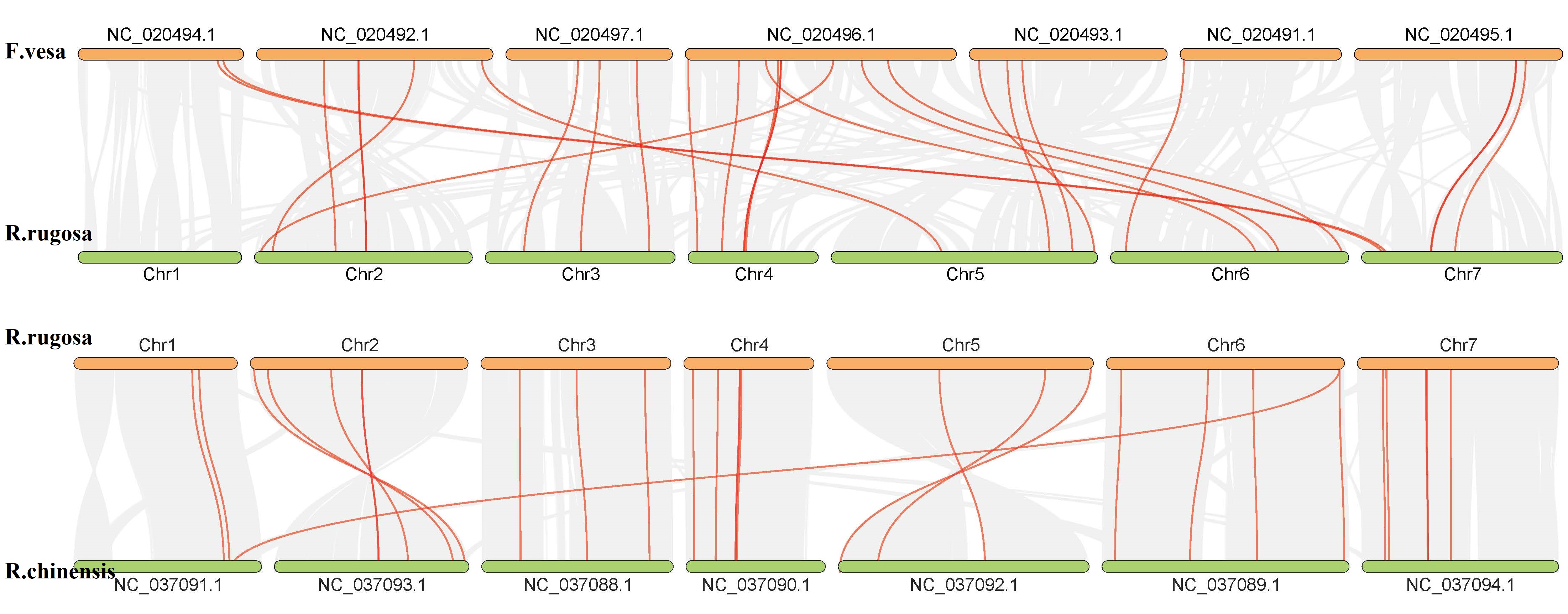

Supplement: Supplementary file 1 [file biology-12-00176-s001.zip › biology-2114111-supplementary/Figure S1.jpg]
